# Echocardiography led to the evaluation of cardiopulmonary resuscitation

**DOI:** 10.1186/2036-7902-6-S1-A12

**Published:** 2014-01-31

**Authors:** M Algaba-Montes, A Oviedo-García, JM Alvarez-Franco, A Segura Grau, J Lopez-Libano, N Diaz-Rodriguez, A Rodriguez-Lorenzo

**Affiliations:** 1 Emergency Department, Valme Hospital, Seville, Members of the Working Group of Ultrasound SEMES_Andalucía and SEMERGEN, Spain; 2 Emergency Department, IB-Salut, Ibiza, Member of the Working Group of Ultrasound SEMERGEN, Spain; 3 MFYC, Médico ecografista en Centro Diagnostico Ecográfico y en Unidad de ecografía general del Hospital San Francisco de Asis, Madrid, Spain; 4 Critical Care Department, Miramar Hospital, Mallorca, Member of the Working Group of Ultrasound SEMERGEN, Spain; 5 Primary Care, Barbadás Primary Care Center, Ourense. Member of the Working Group of Ultrasound SEMERGEN, Spain; 6 Radiology Department, Perpetuo Socorro Hospital, Vigo, Member of the Working Group of Ultrasound SEMERGEN, Spain

## Background

The cardiopulmonary arrest (CA) is a critical situation, where ultrasound is the only diagnostic mode with the possibility of direct action during cardiopulmonary resuscitation (CR) in real time, without interfering with the resuscitation maneuvers. Through a structured process with a focused ultrasound (US) examination procedure conforming to the universal algorithm in cardiopulmonary resucitation, and using the Protocol FEER, it is possible to recognize the relevant pathology and more user-friendly wich cause CA or pulseless electrical activity.

## Objective

To know the utility of ultrasonography in the diagnosis and management of the CA.

## Patients and methods

Literature review of articles published up to December 2011 related to the use of ultrasound as an aid to making decisions and diagnosis in the context of a CA. Key words: Focused echocardiography evaluation resuscitation. Critical care ultrasound.

## Results

The FEER Protocol is a procedure that consists of 10 steps, and that must be run simultaneously during the cycles of CR to reduce interruptions of cardiac massage, with a four chambers subcostal view, and medioclavicular of both hemithorax. Evaluate if there is a cardiac tamponade (CT), severe hypovolemia (SH), pulmonary embolism (PE) or tension pneumothorax (TP).

The absence of cardiac mobility objectified with ultrasound, regardless of whether there is or not electrical activity, is associated with refractoriness to the manoeuvres of cardiopulmonary resuscitation and fatal outcome.

## Conclusion

US during CR reduces the time required to determine the cause of cardiac arrest (CT, SH, PE, TP), and thus to decrease the time until an effective treatment, in addition to detecting whether or not mechanical activity in the heart and distinguish true pulseless electrical activity.
